# Editorial: Type 2 diabetes and cancer: clinical and molecular interactions

**DOI:** 10.3389/fmed.2023.1345732

**Published:** 2024-01-08

**Authors:** Mariachiara Santorsola, Michele Caraglia, Alessandro Ottaiano

**Affiliations:** ^1^Department of Abdominal Oncology, Istituto Nazionale dei Tumori di Napoli, IRCCS “G. Pascale”, Naples, Campania, Italy; ^2^Department of Precision Medicine, University of Campania Luigi Vanvitelli, Caserta, Campania, Italy

**Keywords:** diabetes, genetics, cancer, *p53*, lung cancer, glioma, head and neck cancer

The intricate interplay between Type 2 Diabetes (T2D), impacting over 100 million individuals in Western countries, and cancer, remains poorly understood. The objective of this Research Topic was to investigate and emphasize recent developments in elucidating this complex relationship.

Our group, Ottaiano et al., investigated the connection between metastatic colorectal cancer (CRC) and T2D, hypertension (HT), overweight, and *p53* gene mutations. We hypothesized that specific molecular signatures could partially explain the prognostic course in these patients. Notably, T2D patients exhibited lower responsiveness to first-line chemotherapy and a poorer prognosis compared to non-diabetic counterparts. The co-occurrence of T2D, HT, and overweight was identified as a strong negative independent variable, emphasizing the need for a comprehensive understanding of these interconnected conditions. Mutations in the *p53* gene were associated with weight, and *p53* mutated patients presented a worse prognosis. Mechanistically, insulin resistance, chronic inflammation, and a higher prevalence of *p53* mutations in T2D patients may contribute to unresponsiveness and unfavorable prognosis. The study highlighted the potential impact of therapeutic strategies targeting insulin resistance and inflammation in restoring chemotherapy sensitivity and improving cancer-specific survival in T2D patients. Furthermore, it is crucial to underscore and engage in a discussion with the readers of this editorial, who can be motivated to delve deeper. This relates to the observation that several genes, marked by either mutations or polymorphic variants in T2D, may also contribute to creating a conducive genetic background for CRC. This perspective represents the future for scientifically contextualizing the relationships between cancer and T2D. In particular, some of these genes involved in the phenotypic determinism of T2D are also genes altered in CRC (i.e., *CCND2, CDKN1B, CDKN2A, CDKN2B, EML4, HNF1A, ID3, IGF1, IGF1R, IGF2, INHBA, INSR, IRS1, IRS2*, and *TCF7L2*). An analysis using the Phenolyzer tool is reported in Figure 1. Among these genes, *IGF1R, CDKN2A, CDKN2B, CDKN1B, TCF7L2*, and *IGF1* stand out as the most significant.

**Figure 1:**
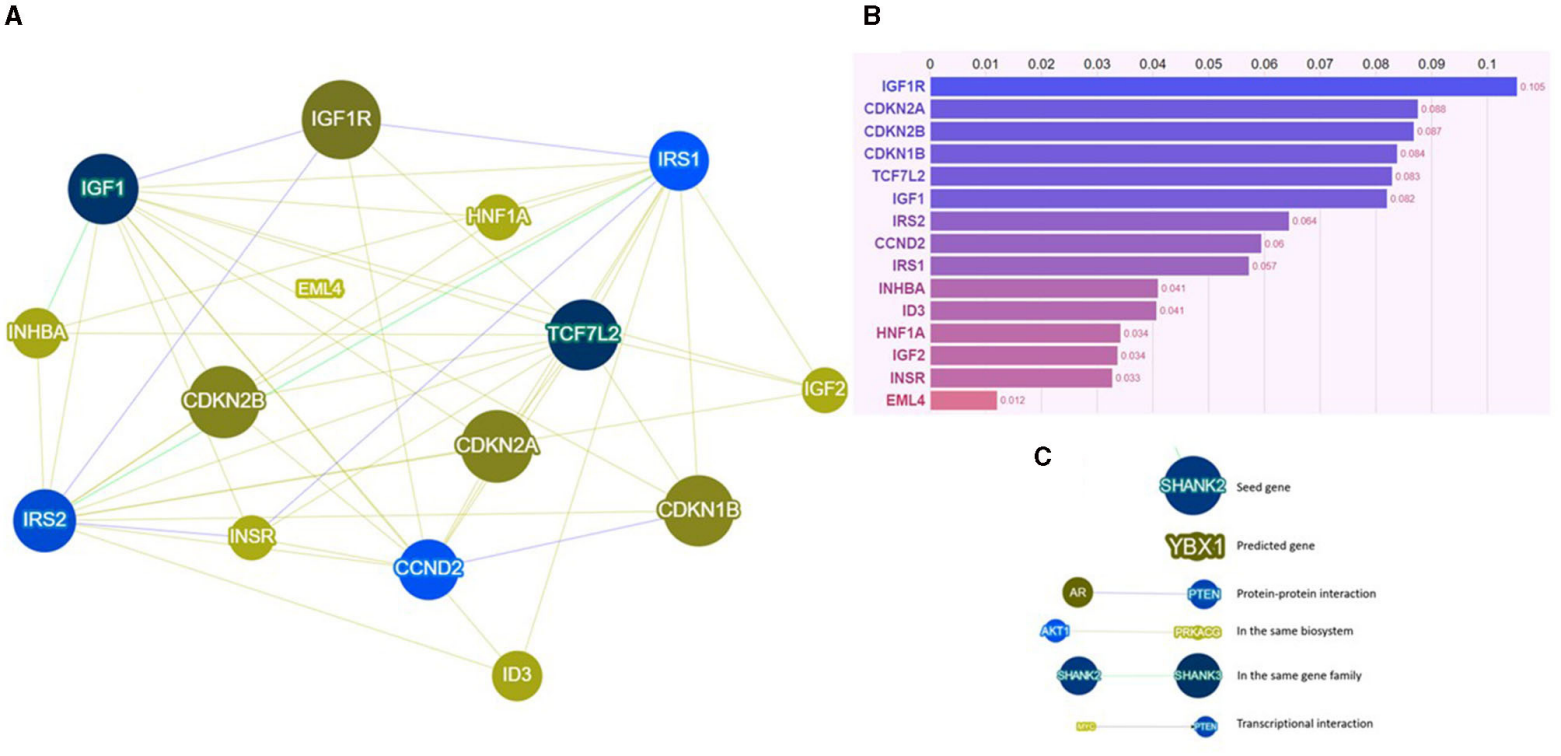
**(A)** Network visualization image, **(B)** results of the score system, and **(C)** legend for the network visualization image of Phenolyzer based on specific interactional contexts. For further insight, visit https://phenolyzer.wglab.org/. Phenolyzer systematically examines key gene-disease databases, including OMIM, Orphanet, ClinVar, Gene Reviews, and GWAS Catalog. It prioritizes genes by considering current scientific knowledge such as shared biological pathways, gene families, gene-gene transcriptional regulation, and protein-protein interactions. The outcomes are presented through a scoring system (visible at the end of each bar in the graph) and a network visualization, offering readers an intuitive overview of the weighted interactional context.

In April 2023, Kim et al. conducted a retrospective study investigating the association between T2D and lung cancer risk in chronic obstructive pulmonary disease (COPD) patients. The analysis revealed an increased risk of lung cancer in COPD patients with T2D, especially among smokers and those in rural areas. Early diagnosis of lung cancer is a fundamental goal capable of significantly impacting mortality attributable to this condition. Chronic inflammation in T2D and COPD was proposed as a contributing factor. The study adds an interesting layer to our understanding, emphasizing the need for heightened vigilance in monitoring lung cancer risk in COPD patients with T2D, especially among specific subgroups. This prompts a reconsideration of clinical strategies for early detection and intervention.

The study conducted by Liu et al. employed Mendelian Randomization to address inconsistencies in prior research regarding the association between adiposity, diabetes, lifestyle factors, and the risk of gliomas. The findings rejected causal links between adiposity, T2D, smoking, alcohol consumption, or coffee intake and glioma development. Communicating negative results in oncology is crucial to address publication bias, ensuring a balanced scientific literature. Such findings also enhance the overall knowledge base by highlighting ineffective approaches, contributing to evidence-based medicine, and elevating research quality.

In the research conducted by De Falco et al., diabetic patients with recurrent or metastatic head and neck cancer demonstrated better progression-free survival and overall survival, even after excluding other clinical confounding factors. The authors postulated a mechanism implicating a modulation in the dynamics of Insulin-like Growth Factor-1 (IGF-1) and the utilization of metformin, exerting a favorable influence on tumor growth. The acknowledged role of metformin as a radiosensitizer in various cancer types prompts the need for a more in-depth investigation into its impact on observed outcomes in head and neck cancer. These findings open avenues for reevaluating treatment strategies in diabetic patients with head and neck cancer. The suggested mechanisms involving IGF-1 and metformin provide a basis for further exploration, potentially influencing therapeutic approaches and patient outcomes.

We express gratitude to the Authors of these articles for contributing to a debate and scientific exploration that is still entirely open and warrants further scientific efforts to unveil epidemiological, biological, and prognostic connections between T2D and tumors. This represents an extremely fascinating field within oncology, holding the potential for advancements that can lead to innovative treatments and precision medicine approaches for oncologic patients with diabetes.

## Author contributions

MS: Methodology, Software, Validation, Writing—review & editing. MC: Conceptualization, Methodology, Validation, Writing—original draft. AO: Conceptualization, Methodology, Supervision, Validation, Writing—original draft.

